# Protocol to characterize longitudinal gut motility in mice using transit time, tissue harvest, and whole-mount immunostaining

**DOI:** 10.1016/j.xpro.2025.103761

**Published:** 2025-04-15

**Authors:** Mary E. Frith, Purna C. Kashyap, David R. Linden, Eugene B. Chang

**Affiliations:** 1 Interdisciplinary Scientist Training Program, University of Chicago, Chicago, IL 60637, USA; 2 Department of Medicine, University of Chicago, Chicago, IL 60637, USA; 3 Division of Gastroenterology and Hepatology, Department of Medicine, Mayo Clinic, Rochester, MN 55905, USA; 4 Enteric Neuroscience Program, Department of Physiology and Biomedical Engineering, Mayo Clinic, Rochester, MN 55905, USA

**Keywords:** Metabolism, Model Organisms, Systems biology

## Abstract

Transit time is a key *in vivo* metric of gastrointestinal (GI) motility, which is a physiologic readout of cellular communication within the enteric system. Here, we present a protocol to characterize longitudinal gut motility in mice. We describe steps for transit testing, whole-mount immunostaining, and tissue harvest. We then detail procedures for image processing and manual cell counting. This protocol seeks to minimize inter-trial variability while assessing cellular and molecular features that may underpin motility differences between experimental conditions.

For complete details on the use and execution of this protocol, please refer to Frith et al.^1^

## Highlights

• Guidance for reproducible *in vivo* gastrointestinal motility studies in mice
• Workflow for gastrointestinal tissue harvest for multiple downstream applications
• Whole-mount staining of enteric neurons and glia for confocal imaging of myenteric ganglia

## Abstract (editor highlights)

Publisher’s note: Undertaking any experimental protocol requires adherence to local institutional guidelines for laboratory safety and ethics.

## Graphical abstract

**Figure:**
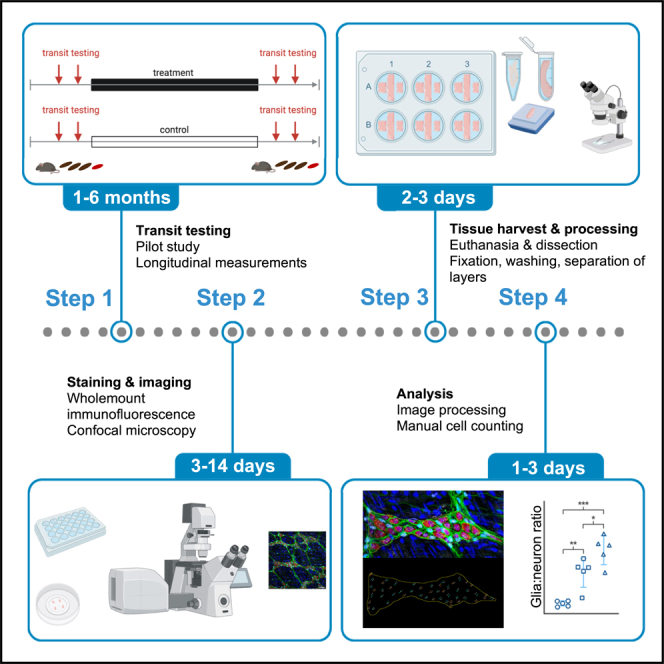

## Before you begin

The protocol below describes the specific steps for assessing gastrointestinal (GI) motility in mice during, before, and after experimental manipulations, followed by tissue harvest and whole-mount immunostaining for downstream tissue analysis.

Whole-gut transit testing (WGTT) is a widely used method of assessing motility in animals, including humans. We have found that there are nuances in conducting WGTT that aid in accuracy and reproducibility but that may be too specific to be outlined in a research paper methods section. Motility is affected by a vast array of factors, including time of day, anxiety level, food intake, and microbiome, so it naturally varies somewhat from day to day. However, WGTT in motility studies is often assessed at a single point in time, leading to variable results that are difficult to compare across studies and experiments. For this reason, and because it is often desirable to understand changes to GI motility longitudinally within the same individual, there is value to testing more than once per each experimental timepoint, to take an average for that time point that more accurately represents the animal’s motility. Our hope is that this protocol provides a standardized and detailed resource helpful for those conducting transit testing, particularly longitudinally.

Given the essential role of the enteric nervous system (ENS) in the regulation of gastrointestinal motility, it is often of interest at the conclusion of a motility study to perform immunostaining of enteric neurons and glia. Most of the cells of the ENS are located in the myenteric plexus, which is sandwiched between the longitudinal and circular muscle layers of the intestinal wall. Because the plane containing the myenteric plexus is relatively thin (often the thickness of one neuronal cell body) and varies in tissue depth based on the thickness of surrounding tissue, it is necessary to perform whole-mount staining to see more than just a few cells of interest in a given section. This method differs from traditional thin-section immunostaining and may be difficult to do successfully without specific instructions, which we will share here. We also seek to describe a workflow that improves organization and reduces time spent troubleshooting.

Conducting an initial pilot study using this pipeline is strongly recommended. This will provide more comfort in handling mice for this type of experiment as well as in establishing clear rules for what constitutes a “red” pellet. Mice also may behave differently in the hands of an inexperienced (and potentially nervous) experimenter than someone more comfortable with the procedures. Practicing the tissue harvest and staining techniques in advance of a larger follow-up study will also help to hone the workflow and maximize efficiency and utility of the follow-up study.

### Institutional permissions

Any experiments must be performed in accordance with relevant institutional and national guidelines and regulations. The experiments described here were approved by the University of Chicago Institutional Animal Care & Use Committee (IACUC). The authors remind the readers to acquire permissions from the relevant institutions before beginning these experiments.

### Preparation for transit testing

**Timing: variable**

***Note:*** Prepare all solutions as described in recipes in the materials and equipment section.

**Figure 1:**
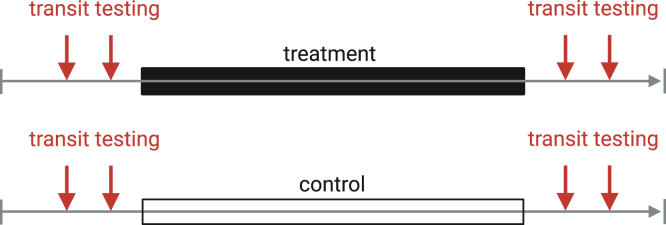
Example timeline for transit testing

***Note:*** Designate general intervals in time for frequency of measurement. When planning, note the “target day”, e.g. when the mouse is exactly a certain age, and plan transit testing dates around that – e.g. 2 days before, 2 days after. Having a range of ∼5 days around the target date allows to test multiple mice at once even if slightly different in age.

***Note:*** Conducting an initial pilot study to gauge sample sizes for sufficient power is strongly recommended.

**Figure 2:**
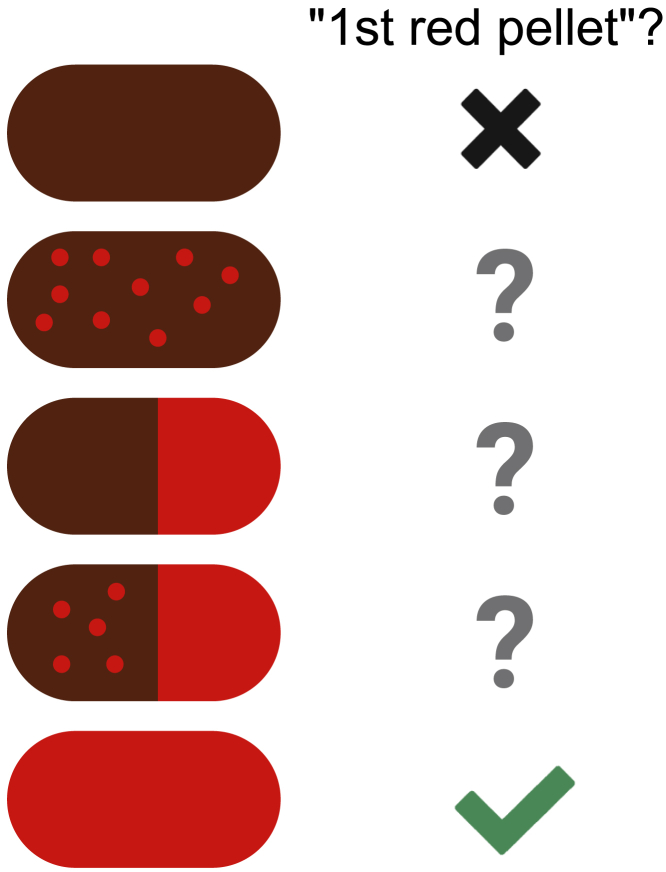
Considerations for defining “red” pellet

***Note:*** We recommend a red pellet be defined as a “fully red pellet”; that is, not one with red specks against a brown background nor one only partially saturated with red dye.

**Figure 3:**
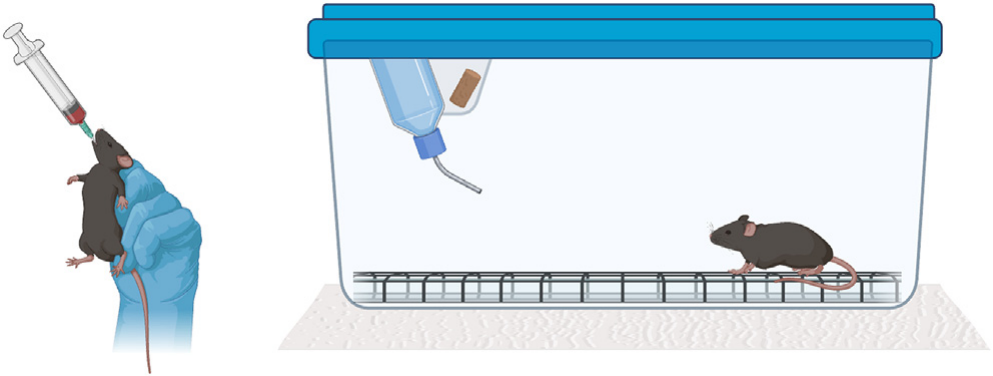
Transit testing setup

1. Carefully plan out timeline for experimental manipulations, measurements, and endpoints (example timeline in Figure 1).

2. Prepare 6% carmine dye solution (materials and equipment setup).
  a. Make 0.5% methylcellulose solution.
    i. Heat 10 mL water in glass beaker to at least 80°C using a microwave or heat block.
      **CRITICAL:** Use caution with hot glass.
    ii. Add methylcellulose powder to hot water with light agitation.
    iii. Agitate mixture until particles are evenly dispersed.
    iv. Add 20 mL volume of water as cold water or ice to lower the temperature and increase viscosity.
      ***Note:*** Cool solution to 0°C–5°C for 20–40 min.
    v. Continue agitation for at least 30 min after proper temperature reached.
  b. Add 1.8 g carmine powder.
    i. Use caution when weighing powder, as it is lightweight and easily blown across workspace (carmine powder is non-toxic).
    ii. Add carmine powder to the cooled methylcellulose solution and agitated at 18°C–25°C until evenly dispersed (e.g., 30 min).
  c. Sterilize the solution by autoclaving (liquid cycle).
    **CRITICAL:** Make sure the vessel is at least 2.5× the volume of the solution to prevent boiling over. If using a bottle, **do not tighten the lid** before autoclaving.
    ***Note:*** Vortex aliquots on day of testing, as dye may have partially separated.
3. If fecal collection will be performed (for fecal water fraction analysis and/or microbiome profiling), prepare two labeled microcentrifuge tubes per animal (Optional).
  a. Weigh one of the two tubes in advance using an analytic balance and record this number for later reference.
4. Define clear criteria for a “positive” / “first” red pellet (Figure 2).

5. Set up for transit testing (Figure 3).
  a. Place one standard clean mouse cage per mouse on white paper inside a biological safety cabinet.
    ***Note:*** The cages should be empty, without bedding.
    i. Place a stainless steel raised floor cage insert inside each empty cage.
    ii. Prepare a food/water hopper and a lid for each cage.
    iii. Gather one water bottle per cage.
      ***Note:*** After the animal is placed into the cage for transit testing, the water bottle and 1–2 food pellets may be placed in the hopper.
      ***Note:*** The steel rack cage insert is used such that stool pellets will fall through the grating, out of reach of the mouse. If an appropriately sized rack cannot be found for the cages available at one’s institution, it may be necessary to source cages of a different size specifically to be used for transit testing. The mouse is returned to its home cage after transit testing.

### Preparation for tissue harvest

**Timing: 4–5 days**

6. Prep silicone plates
  a. Sylgard 184 Elastomer Kit: Per the manufacturer’s instructions, mix the curing agent and base.
  b. Distribute the liquid silicone agent into several 6-well plates, 2 medium petri dishes, 1 large petri dish, at least 1 12-well plate, and optionally, 1 24-well plate.
    i. Dispense enough silicone to cover the bottom of each well/dish (at least 0.5 cm depth) (e.g., ∼25 mL total per each 6-, 12-, or 24-well plate or 10 cm petri dish; ∼35 mL total per each 15 cm petri dish).

***Note:*** The total number of wells across the 6-well plates should be at least equal to the number of mice to undergo tissue harvest.

**CRITICAL:** Using the plates before they have cured for 4–5 days may result in the silicone detaching from the plate.

**Pause point:** Cover dishes with lids and allow to cure for 4–5 days at 18°C–25°C.

**Figure 4:**
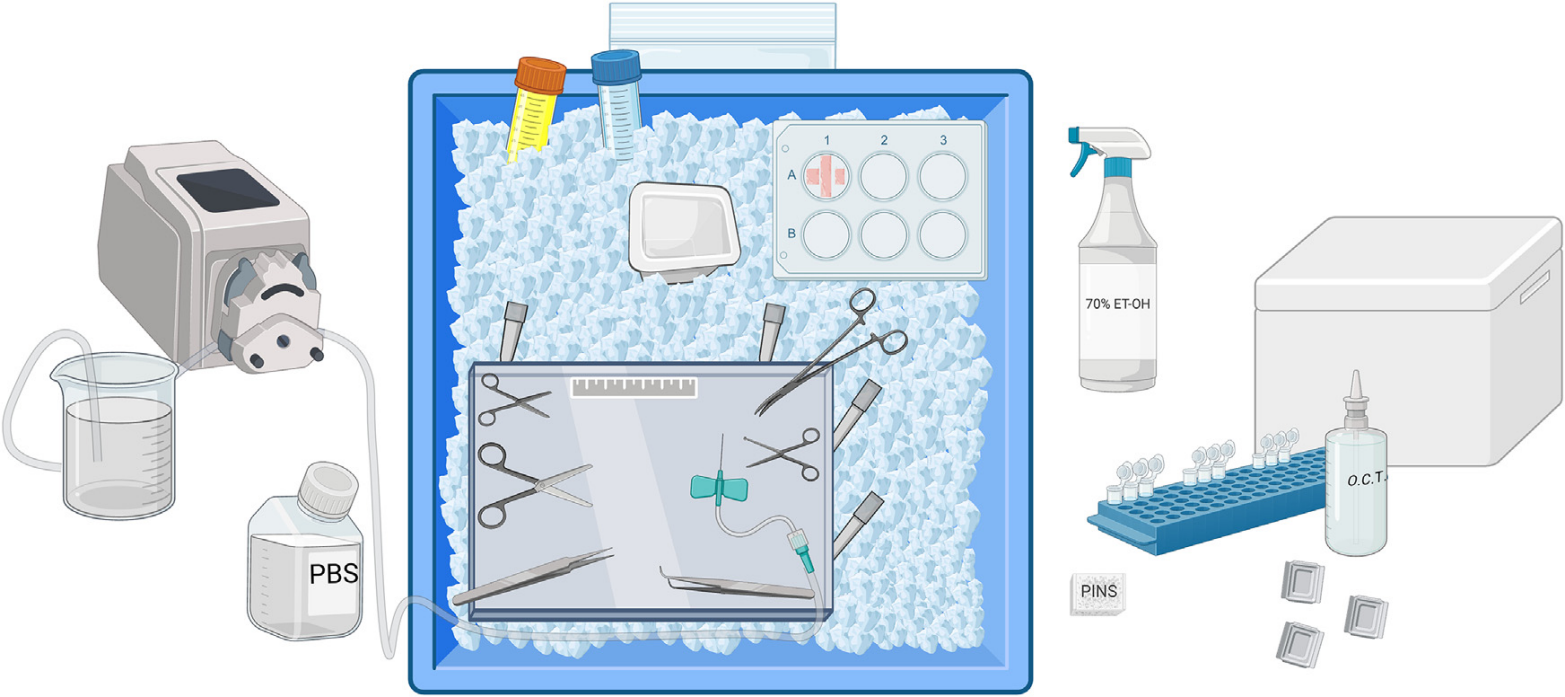
Tissue harvest setup Items include ice bin with ice, glass dissection plate, 6-well plate with silicone, 4 large pipette tips (to stabilize glass plate), ruler, tools, peristaltic pump and associated tubing, beaker, PBS, butterfly needle, Zamboni fixative and 4% PFA in PBS in conical tubes, weigh boat containing PBS, O.C.T., tissue embedding molds, pins, microcentrifuge tubes containing RNAlater or 40% glycerol in PBS, bag for disposing mouse carcasses, and foam box containing dry ice.

7. Set up for tissue harvest.
  a. Gather supplies and reagents as diagrammed in Figure 4.
    i. Fill ice bin with ice.
    ii. Place dry ice in foam box.

**CRITICAL:** Do not use anodized pins in place of stainless steel pins as anodized pins may interact with tissue processing solutions.

8. Ensure availability of required equipment, including dissection hood if using PFA or other substances with fumes or if required due to pathogens in the mice.
9. Label tubes, plates, and embedding mold according to the tissues to be collected.

***Note:*** We usually use 2 microcentrifuge tubes containing RNAlater each for small and large intestine, and another tube with glycerol solution for cecum.

10. Prepare pump with tubing, and prime pump, if transcardial perfusion will be performed.
  a. Set the pump to a rate of 3–5 mL/min.^2^^,^^3^
11. Prepare PBS w/ 40% glycerol, phosphate buffer, Zamboni fixative, PBS with 10% sucrose, and PBS with 20% sucrose / 10% glycerol as indicated in materials and equipment Setup.

### Preparation for whole-mount immunostaining

**Timing: variable**

12. Prepare solutions, gather supplies, and ensure equipment availability.
  a. Prepare SuperblockT, PBST, and Tris-EDTA buffer as indicated in materials and equipment Setup.

***Note:*** This protocol uses HuCD, GFAP, and Sox10 primary antibodies and Alexa Fluor secondary antibodies (key resources table).

***Note:*** Solutions for M.O.M. Blocking, antibodies, and nuclear stain may be prepared immediately before they are to be used during whole-mount staining.

***Note:*** Supplies for this step include 12- or 24-well silicone plates (prepared in Preparation for Tissue Harvest), Stainless Steel Minutien Pins (0.2 mm diameter), and glass bottom dishes.

13. Download image processing software Fiji/ImageJ.

***Note:*** Fiji/ImageJ is free and available for download online (key resources table).

14. Identify tissue to be used for staining controls (i.e., tissue of the same type as experimental tissues).
15. Separate muscularis propria from epithelium under dissecting microscope.
  a. Add a small amount of PBS to a large plate containing silicone, enough to cover the tissue (e.g., 3 mL).
  b. Use pins to secure the tissue to the plate, with mucosal side facing up.
  c. Using the dissection microscope, zoom in and focus to sufficiently visualize the division of the mucosa and muscularis propria.
  d. Gently grasp the muscularis layer with curved fine forceps and the mucosal layer with fine forceps, carefully separating the two layers.
    ***Note:*** It is okay if the epithelium tears for the purposes of this protocol; the priority is maintaining the integrity of the muscularis propria.
    ***Note:*** If unable to peel apart the two layers, it may be helpful to scrape the mucosal layer gently off of the muscularis in one area to find the plane to facilitate the separation of the layers.
  e. Once the plane between the muscularis propria and mucosa has been established, hold the muscularis propria down against the silicone gently with the curved side of curved forceps while carefully peeling away the mucosal layer.
    ***Note:*** Separating intestinal layers can be a slow process but gets faster with practice.
16. Label glass bottom well plates.

## Key resources table

**Table:** 

REAGENT or RESOURCE	SOURCE	IDENTIFIER
**Antibodies**		

Mouse monoclonal anti-HuC/HuD (2 μg/mL)	Invitrogen	Cat#A-21271
Chicken polyclonal anti-GFAP (1:2,000)	Abcam	Cat#ab4674
Goat polyclonal anti-Sox10 (1:500)	R&D Systems	Cat#AF2864
Donkey anti-mouse Alexa Fluor 555 (1:500)	Invitrogen	Cat#A-31570
Donkey anti-chicken Alexa Fluor 488 (1:500)	Invitrogen	Cat#A78948
Donkey anti-goat Alexa Fluor 647 (1:500)	Invitrogen	Cat#A-21447

**Biological samples**		

Mouse stool	Frith et al.^1^	N/A

**Chemicals, peptides, and recombinant proteins**		

Carmine	Sigma-Aldrich	Cat#C1022
Methyl cellulose	Sigma-Aldrich	Cat#M0512
RNALater Stabilization solution	Invitrogen	Cat#AM7024
MOM Blocking Reagent	Vector Labs	Cat#MKB-2213-1
Prolong Gold Antifade mountant	Invitrogen	Cat#P36930
Sylguard Elastomer Kit	Electron Microscopy Sciences	Cat#24236-10
SuperBlock blocking buffer	Thermo Scientific	Cat#PI37515
Picric acid, saturated aqueous, approx. 1.2% (w/v)	Ricca	Cat#586016
Paraformaldehyde solution, 4% in PBS	Thermo Scientific	Cat#AAJ19943K2
Sodium phosphate monobasic (NaH_2_PO_4_)	Sigma-Aldrich	Cat#S3139
Sodium phosphate dibasic (Na_2_HPO_4_)	Sigma-Aldrich	Cat#S9763
Phosphate-buffered saline (PBS)	Thermo Fisher Scientific	Cat#10010023
Glycerol	Sigma-Aldrich	Cat#G7893
PBS w/ 0.1% NaN_3_	Alkali Scientific	Cat#PBF500
Tris Base	Sigma-Aldrich	Cat#T6687
EDTA	Sigma-Aldrich	Cat#E6758
Tween 20	Sigma-Aldrich	Cat# P9416
Triton X-100	Sigma-Aldrich	Cat#X100
Sucrose	Sigma-Aldrich	Cat#S5016
70% Ethanol	Sigma-Aldrich	Cat#65350-M
O.C.T. Embedding Medium	Fisher	Cat#23-730-571
Hoechst 33342	Fisher	Cat#BDB561908
Sodium hydroxide solution (for pH titration)	Sigma-Aldrich	Cat#1091371000
Hydrochloric acid solution (for pH titration)	Sigma-Aldrich	Cat#13-1700

**Experimental models: Organisms/strains**		

Mouse: C57Bl/6, male or female ≥3 weeks of age	Bred in-house	RRID:MGI:2159769

**Software and algorithms**		

Fiji/ImageJ	Schindelin et al.^4^	https://fiji.sc/

**Other**		

35 mm Glass bottom dish with 20 mm micro-well #1.5 cover glass	Cellvis	Cat#D35-20-1.5-N
Stainless Steel Minutien Pins with 0.2 mm diameter	Fine Science Tools	Cat#26002-20
Stainless Steel Raised Floor Cage Inserts	Ancare	Cat#N10SSRWF
Mouse cage, wire bar lid, water bottle, filter top	Allentown (or per institution standard)	PC75JHT, WBL1019RMB, PC15/17BHT-85 MBT7115RHHR
Rectangular ice bin	Fisher	Cat#07-210-093
Peristaltic pump (capable of 1–10 mL/min)	Fisher	Cat#13-200-001
Tubing for peristaltic pump	Fisher	Cat#14-170-11E
Glass dissection plate	Chemglass	Cat#CG-1904-17
4 large pipette tips	Fisher	Cat#05-403-51
6-well plates	Fisher	Cat#07-200-80
Weigh boat	Fisher	Cat#01-549-753
Tube rack	Fisher	Cat#03-411-834
Bag for mouse carcass disposal	Fisher	Cat#01–816B
Ruler	Fisher	Cat#12-000-152
Benchtop orbital shaker	Fisher	Cat#15-600-321
500 mL beaker	Fisher	Cat#31-502-281
12-well plates	Fisher	Cat#07-200-81
24-well plates	Fisher	Cat#07-201-590
Tissue embedding molds	Fisher	Cat#22-363-554
Foam box for dry ice	Fisher	Cat#50-339-43
Microcentrifuge tubes	Fisher	Cat#02-681-332
Curved fine forceps	Fine Science Tools	Cat#11370-31
Straight fine forceps	Fine Science Tools	Cat#11254-20
Straight forceps	Fine Science Tools	Cat#11018-12
Long forceps	Fisher	Cat#S08102
Surgical scissors	Fine Science Tools	Cat#14001-12
Fine scissors	Fine Science Tools	Cat#14083-08
Ball tip scissors	Fine Science Tools	Cat#14086-09
Dissection scissors with rounded edge	Fine Science Tools	Cat#14084-09
Hemostat	Fine Science Tools	Cat#13015-14
Butterfly needle with tubing	Fisher	Cat#22-024-241
Plastic transfer pipette	Fisher	Cat#13-680-50
Confocal microscope	Leica	leica-tcs-sp8-x
Laboratory oven	Fisher	Cat#NC1695553
Dissection/stereo microscope	Evident Scientific	SKU#szx7
Analytical balance	Fisher	Cat#01-671-309
Vortex	Fisher	Cat#02-215-414
pH meter	Fisher	Orion Lab Star PH111
50 mL conical tubes	Fisher	Cat#14-432-22

Please note that many of the resources in this table may be obtained from a variety of sources; the ones listed are simply examples.

## Materials and equipment

**Table:** Carmine red dye

Reagent	Final concentration	Amount
Carmine powder	6% (w/v)	1.8 g
Methylcellulose	0.5% (w/v)	150 mg
Water	N/A	30 mL
**Total**	**N/A**	**30 mL**

Store at room temperature for up to 6 months.

***Alternatives:*** While carmine dye has been used to study transit testing for more than a century,^5^^,^^6^ so have other dyes, particularly Evan’s blue^7^ and charcoal.^6^^,^^8^ FITC-dextran is less suitable for this protocol as it is not inert (i.e., may enter the bloodstream based on intestinal permeability), requires fluorometry, and is generally used as a terminal assay (i.e., mice are euthanized immediately to facilitate transit analysis).^9^

**Table:** 1 M phosphate buffer pH 7

Reagent	Final concentration	Amount
Sodium phosphate monobasic (NaH_2_PO_4_)	0.39 M	23.25 g
Sodium phosphate dibasic (Na_2_HPO_4_)	0.61 M	43.45 g
Water	N/A	500 mL
Total	1 M	500 mL

Filter sterilize with a 0.22 μm filter. Store at room temperature for up to 12 months.

**Table:** Zamboni fixative

Reagent	Final concentration	Amount
4% PFA in PBS	0.5%	2.5 mL
1 M Phosphate Buffer pH 7	100 mM	2 mL
Picric acid saturated aqueous solution (∼1.2%)	∼0.18%	3 mL
ddH_2_O	N/A	12.5 mL
Total	N/A	20 mL

Prepare fresh on the day of use.

**CRITICAL:** Please note that the aqueous form of picric acid (∼1.2% in water) is distinct from (and less hazardous than) pure solid picric acid, which is explosive. Nonetheless, the saturated aqueous solution is an irritant and may become explosive if dried, so should be handled with caution. Use a chemical fume hood with gloves, lab coat, and eye protection. Keep away from flame. Do not allow solution to dry. Dispose this all and other solutions appropriately, in accordance with institutional and regional regulations.

***Alternatives:*** 4% PFA in PBS or 10% neutral buffered formalin may also work, but we have not extensively evaluated these for this specific application.

**Table:** Tris-EDTA pH 9.0 with 0.05% Tween 20

Reagent	Final concentration	Amount
Tris Base	10 mM	1.21 g
EDTA	1 mM	0.37 g
ddH_2_O	N/A	999.5 mL
Sodium hydroxide 1N	N/A	as needed
Hydrochloric acid (HCl) 1 M	N/A	as needed
Tween 20	0.05%	0.5 mL
Total	N/A	1000 mL

Store at room temperature for up to 3 months or at 4° C for up to 1 year.

**CRITICAL:** Sodium hydroxide and HCl are corrosive. Use under a chemical fume hood wearing gloves and eye protection.

***Alternatives****:* Citrate buffer pH 6.0 may work better for certain antibodies. Pre-made Tris-EDTA buffer solutions are also commercially available.

**Table:** PBS w/ 0.3% Triton X-100 (PBST)

Reagent	Final concentration	Amount
PBS	99.7%	997 mL
Triton X-100	0.3%	3 mL
Total	N/A	1000 mL

Store at room temperature for up to 3 months or at 4°C for up to 1 year.

**Table:** Superblock w/ 0.3% Triton X-100 (SuperblockT)

Reagent	Final concentration	Amount
Superblock (PBS)	N/A	498.5 mL
Triton X-100	0.3%	1.5 mL
Total	N/A	500 mL

Store at 4°C for up to 1 year.

**Table:** Cecal preservation solution (40% glycerol in PBS)

Reagent	Final concentration	Amount
PBS	N/A	60 mL
Glycerol	40%	40 mL
Total	N/A	100 mL

Store at room temperature for up to 12 months or at 4°C for up to 2 years.

**Table:** PBS with 10% (w/v) sucrose

Reagent	Final concentration	Amount
PBS	N/A	500 mL
Sucrose	10%	50 g
Total	N/A	500 mL

Filter sterilize with a 0.22 μm filter and store at 4°C for up to several months.

**Table:** PBS with 20% (w/v) sucrose / 10% glycerol

Reagent	Final concentration	Amount
PBS	N/A	450 mL
Sucrose	20%	100 g
Glycerol	10%	50 mL
Total	N/A	500 mL

Filter sterilize with a 0.22 μm filter and store at 4°C for up to several months.

## Step-by-step method details

### Longitudinal assessment of gastrointestinal motility

**Timing: 3–12 h per experimental session; 1–6 months total**

Transit testing provides a readout of gastrointestinal motility *in vivo*. It can be repeated in the same animal to assess the effect of experimental manipulations on motility.

***Note:*** It is best to perform transit testing at the approximately the same time of day in all groups, as motility patterns may differ across the day.

***Note:*** It is recommended to test each animal at least 2× for each time point (separated by several days) to allow an average to be computed for that mouse.

1. Transfer mouse from home cage to test cage, gavage with dye, and record the gavage time.
  a. Scruff mouse, check ear tag number if applicable.
  b. Gently allow the gavage needle to slide down the the back of the tongue into the esophagus of the mouse.
  c. Gently administer 150 μL carmine dye and remove the needle.
  d. Place the mouse inside the transit testing cage.
  e. Place the food hopper on top, add 1–2 food pellets, and add a water bottle.
  f. Cover the cage with a lid.

***Note:*** Consider using different gavage needles and carmine dye aliquots for different groups.

2. If appropriate for your study, collect two stool pellets from each mouse using long forceps (Optional).
  ***Note:*** One fecal pellet is for downstream microbiome analysis, and the other is for fecal water fraction.
  a. Place each pellet in its own capped tube, including one of the labeled pre-weighed tubes from the “preparation for transit testing” step.
  b. Store the pellet for microbiome analysis at −20° to −80°C as soon as possible after collection prior to DNA extraction.
    ***Note:*** For the pellets collected for fecal water fraction analysis, avoid collecting pellets saturated by urine or water, and avoid letting the pellets dry before measuring wet weight.
    ***Note:*** It is best to collect the first 2 fecal pellets as soon as possible after they are excreted. This helps to ensure the pellet is normal in color at baseline. The quality of the microbial DNA may also decrease if collection is delayed.
  c. Weigh the tube + wet pellet together and record the mass.
    i. Subtract the empty tube mass to obtain the “wet pellet” mass.
  d. Open the tube to air and place the open tube and pellet into an oven set to 55°C.
  e. Let pellet dry in the oven at least overnight, and no more than 4 days.
  f. Cap the tube and re-weigh the tube and dry pellet, recording the mass.
    ***Note:*** In our experience, the tube mass does not change with drying, but if you are uncertain, it is recommended to re-weigh the empty tube after discarding the dry pellet or to directly measure the mass of the dry pellet.
  g. Calculate the fecal water fraction as 1-(dry weight/wet weight).
3. Beginning around 1 h after the gavage time, monitor the mice as continuously as possible.
  ***Note:*** We advise monitoring the mice more closely while they are actively moving around in the cage, as it is easy to miss a pellet that the mouse eats immediately after excreting.
  ***Note:*** An alternative approach to continuous monitoring is to check for red pellets at predetermined intervals, such as every 15 min after the first 1 h.
  a. Keep a running count of any pellets eaten by the mouse and/or removed from the cage to ensure an accurate pellet count later.
  b. If a pellet looks like it might be red, remove it from the cage and smear onto white paper.
  c. Remove pellet gently using long forceps, trying to minimize disruption to the animal.
  d. Identify based on your pre-determined rule whether the pellet is the “first” red pellet.
    ***Note:*** See note in “Preparing for transit testing” above about defining a “first red pellet”.
    ***Note:*** If a pellet’s color is ambiguous despite having clear criteria for a “red” pellet, record the time of the possible “red” pellet and then wait for the next one to allow comparison - sometimes by comparison it becomes clear that the second one is “fully red” and the first is “reddish”. Record both times in your notes – i.e. the “reddish” and “full red” times – and consider taking a photo of the pellets for later reference or comparison in future runs for consistency across studies.
4. Once the “first red” pellet appears according to your criteria, record that time.
5. Return the mouse to its home cage.
6. Count the pellets in the experimental cage, noting the number of “brown” and “red” pellets.

***Note:*** These data may be useful later for fecal pellet output assessment.

7. Remove the steel cage insert from the cage.

***Note:*** The cage insert may be washed by hand or in a dishwasher and sterilized in an autoclave if needed before the next experiment.

***Note:*** Consider doing at least 2 runs per mouse per time point, separated by several days to ensure the dye is cleared from the GI tract of the mouse between runs.

***Note:*** If re-testing will be performed in a few days, consider placing the mouse in a fresh cage 1 day after transit testing, to reduce the availability of dye-containing stools in the cage for the mouse to re-ingest prior to the next transit test.

### Tissue harvest for downstream whole-mount staining and sequencing applications

**Timing: 2–3 days**

Tissue harvest from experimental animals at the study endpoint can provide tissue that is suitable for several downstream applications, including whole-mount staining and RNA-based assays. The collection scheme described here can be adapted to meet the needs of your study. For example, consider whether transcardial perfusion is indicated, and if so, which perfusate should be used (i.e., PBS or fixative). Here we provide tips for organizing intestinal samples for whole-mount immunofluorescence, cecal microbiome analysis, and RNA applications. We also preserve a small amount of intestinal tissue for frozen sections in case the need arises.

8. Euthanize animal with CO_2_ asphyxiation or in accordance with approved animal protocols.
9. Transfer mouse to dissection plate over ice.
10. Wet the mouse abdomen with 70% ethanol.
11. Cut the abdominal wall laterally and vertically using surgical scissors and forceps.
12. Perform transcardial perfusion to evacuate blood from the mouse tissues (Optional).
  **CRITICAL:** If transcardial perfusion will be performed, it is important to move quickly but carefully, as transcardial perfusion requires ongoing cardiac contractions.
  a. Lift the sternal cartilage and secure with hemostat to provide access to the diaphragm.
  b. Cut diaphragm, taking care to not cut the heart.
  c. Cut upward toward the axilla through the ribs to allow the anterior chest wall to move away from the heart.
  d. Each in short succession:
    i. Carefully snip the right atrium with scissor.
    ii. Start the pump (rate 3–5 mL/min).
    iii. Insert the butterfly needle into the left ventricle at the heart apex.
      **CRITICAL:** The needle and attached tubing should be primed with PBS beforehand, as noted in Preparation for tissue harvest above.
  e. Hold the needle in place until the fluid flowing from the right atrium is clear.
13. Move intestines to the side to reveal mesenteric lymph nodes.

***Note:*** Mesenteric lymph nodes usually appear as a “string of pearls” along the center of the mesentery.

14. Cut out the mesenteric lymph nodes to allow the bowel loops to move more freely.
15. Cut at the pyloric sphincter.
16. Cut the distal colon at the rectum.
17. Remove the intestine from the mouse from the pyloric sphincter to the distal colon.

***Note:*** It may be necessary to cut and move the gonadal organs to access the most distal part of the colon.

18. Measure the length of small intestine, colon, and cecum.

**CRITICAL:** When measuring, line up the segment along a ruler using minimal tension, which should be consistent across animals.

19. Record these measurements.
20. Gently remove mesenteric fat from the intestine with forceps or fingers.
21. Cut at the ileocecal junction and the junction of the cecum to proximal colon to separate the small intestine, cecum, and colon.

**Figure 5:**
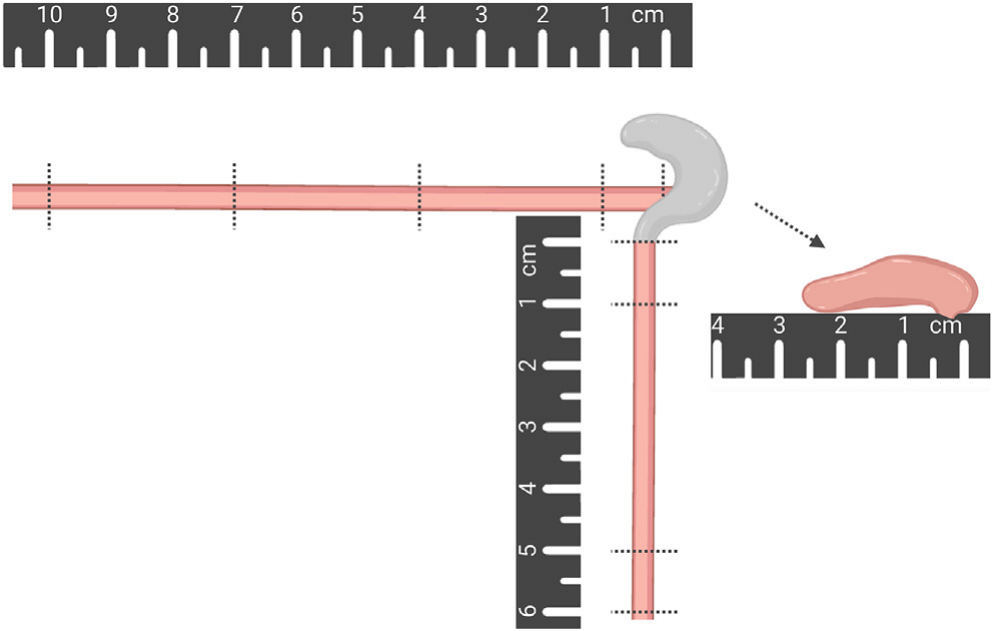
Intestinal section cuts

***Note:*** See diagram of section cuts in Figure 5.

22. Cut the small intestine at a point 10 cm proximal to the cecum.
23. Use the rounded edge of closed dissection scissors to gently push the intestinal contents out of the intestine.
24. Divide that segment of ileum into 4 pieces, cutting 1 cm proximal to the cecal end, 4 cm proximal to the cecal end, and 7 cm proximal to the cecal end.

***Note:*** This yields a 1 cm segment and 3 × 3 cm segments (Figure 5).

25. Place the 1 cm ileal segment into a mold containing OCT and place the mold onto dry ice; it can be used later for frozen tissue sections if needed.
26. Cut open the 1^st^ and 3^rd^ 3 cm ileal segments longitudinally with ball tip scissors.
  a. Rinse the segments in cold PBS.
  b. Place those segments into a microcentrifuge tube containing 600–1,000 μL RNAlater, labeled for ileum.
  c. Do the same for the middle section of the colon.

***Note:*** These tissues may be used in the future for RNA applications.

***Note:*** Tissues in RNAlater can be stored at −20°C in microcentrifuge tubes.

**Figure 6:**
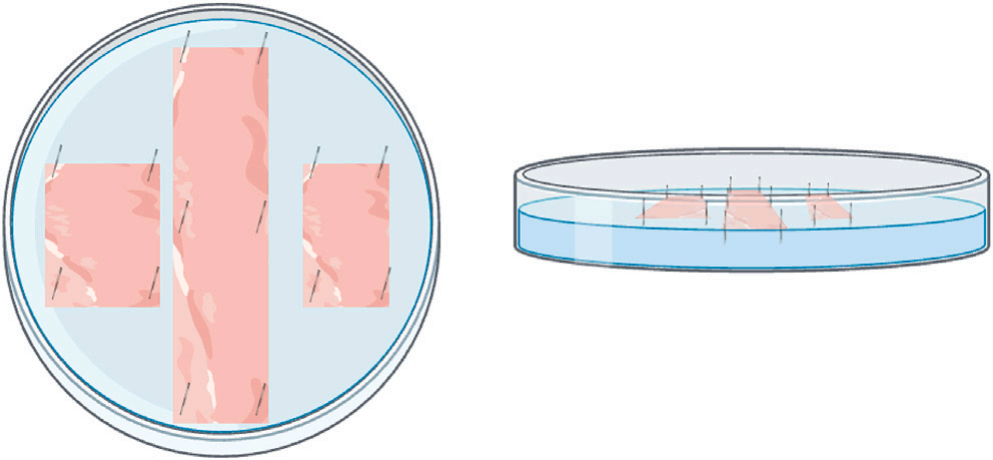
Schematic of tissues preserved for whole-mount immunofluorescence These tissues are pinned in one well of a silicone-containing 6-well plate and include segments of proximal colon, ileum, and distal colon.

27. Open the 2^nd^ 3 cm ileal segment longitudinally with ball tip scissors as was done with the other segments.
28. Rinse in cold PBS.
29 Pin around the tissue edges to flatten the tissue, muscle side down, in the center of the PBS-containing designated well of a silicone coated 6-well plate (Figure 6).

**CRITICAL:** When pinning, aim to create uniform tension throughout the segment (i.e. just loose enough to avoid tearing, but still relatively taught to provide maximum separation between cells for clarity on image analysis).

30. Remove the intestinal contents from the colon in a similar way as for the small intestine (with the rounded edge of closed dissection scissors).
31. Cut the center colon segment longitudinally.
32. Rinse in cold PBS.
33. Place segment into RNAlater.
34. Cut the flanking 1 cm colon segments longitudinally.

**CRITICAL:** Keep track of which segment is proximal versus distal colon.

***Note:*** Proximal and distal colon segments can usually be visually distinguished by luminal diameter (proximal diameter is larger) and by epithelial appearance (proximal has large “V” shaped ridges, while distal epithelial ridges appear more linear).

35. Rinse segments in cold PBS.
36. Cut segments longitudinally.
37. Pin the 1 cm colon segments on either side of the 3-cm ileum segment in the well of the 6-well silicone coated plate.
  a. Pin the ∼1 cm segment of proximal colon (just distal to the cecum) to the left of the 3-cm ileum segment in the silicone dish.
  b. Pin the ∼1 cm segment of the distal-most colon to the right of the 3-cm ileum segment.

**CRITICAL:** Orient tissues in the same way every time and label which tissue piece is from which segment of intestine.

**CRITICAL:** Note the animal ID and date of tissue harvest.

38. Once all three tissue segments are pinned flat in the well, remove the PBS from the well with a bulb suction pipet.
39. Add cold 4% PFA in PBS to the well, enough to cover the tissue (∼2–3 mL).

**CRITICAL:** Use a chemical fume hood when handling 4% PFA.

***Note:*** The use of 4% PFA here is to begin the fixation process (and thus halt tissue degradation) of the flattened tissue segments while the rest of the dissection steps are being completed. The 4% PFA is replaced with Zamboni fixative at the end of dissections.

40. Place the entire cecum, or as much as will fit, into a 2 mL labeled microcentrifuge tube containing 40% glycerol in PBS.
  a. Place that tube onto dry ice.

***Note:*** Later, the tube containing frozen cecum may be transferred to a −80°C freezer for longer term storage.

***Note:*** Glycerol solutions in PBS, such as the one used here, are commonly used to preserve gastrointestinal microbes at low temperatures. However, flash freezing using other methods may be suitable for some studies.

41. For tissues to be used for whole-mount immunofluorescence (i.e., tissues pinned in 6-well plates), replace PFA with Zamboni fixative.

**Pause point**: Let shake gently for 12–48 h at 4°C.

42. Remove fixative solution from wells using a plastic transfer pipette.

**CRITICAL:** Dispose of fixative solutions in accordance with institutional and regional regulations.

43. Wash tissues 3 × 5 min with PBS.
44. Add PBS w/ 10% sucrose.
45. Let shake gently at 4°C for 2–5 h.
46. Remove PBS w/ 10% sucrose.
47. Add PBS w/ 10% glycerol 20% sucrose.

**Pause point:** Let shake gently at 4°C for 12–48 h.

48. Remove PBS w/ 10% glycerol 20% sucrose.
49. Rinse tissues 1× in PBS.
50. Add PBS w/ 0.1% NaN_3_ (key resources table).
51. Seal plate with parafilm.
52. Store plate at 4°C for up to 12 months.

**CRITICAL:** Check plates every month to ensure PBS has not evaporated; replenish if so.

***Note:*** Label tissue storage plates with a designated ID / number. Keep a spreadsheet listing which tissues are on which plate, as it can become cumbersome to sort through stacks of unorganized plates to find tissues from specific animals.

### Whole-mount staining, imaging, and analysis of myenteric neurons and glia

**Timing: 3–14 days**

Whole-mount immunofluorescence staining of intestinal muscularis propria and subsequent confocal microscopy facilitates assessment of spatial relationships between cells of the myenteric plexus and surrounding muscle layers.

53. Separate MP from mucosal layer under dissection microscope if this was not already done (see preparation for whole-mount immunostaining).
54. Organize tissues pinned in 12- or 24-well plate containing PBS.

***Note:*** Generally, a tissue piece of just a few square millimeters is of sufficient size for taking several non-overlapping images with the 20× objective.

55. Replace PBS with Tris-EDTA buffer (materials and equipment setup).
56. Perform heat-induced epitope retrieval (HIER) at 85°–95°C for 15–60 min in an oven.
  a. Cover plate with a lid to reduce buffer evaporation.

**CRITICAL:** Ensure that buffer volume is sufficient such that tissue does not dry out during incubation.

***Note:*** A bead bath could be used instead of an oven if the plate is sufficiently sealed and protected.

***Note:*** HIER in Citrate Buffer pH 6.0 may be more suitable for some antibodies.

***Note:*** As a possible alternative to HIER with buffer, we have also had some success with chemical based epitope retrieval step using 30% 1N potassium hydroxide for 30 min shaking at room temperature.

57. Remove from oven to let tissue cool for 10–20 min.
58. Wash tissues with PBST 3 × 2 min each.
  a. For each wash, agitate tissue on an orbital shaker at 18°C–25°C.
59. Incubate in SuperblockT at 37°C for 5–10 min or at 18°C–25°C for 30 min.
60. Wash 3 × 2 min each in PBST.
  a. Prepare Mouse-on-mouse (M.O.M.) blocking solution by placing 1 drop of M.O.M. blocking reagent per each 2.5 mL PBST.

***Note:*** The recommended volume of solution per well is at least 500 μL per well of a 24-well plate, 1 mL per well of a 12-well plate, and 2 mL per well of a 6-well plate.

61. Incubate tissue in M.O.M. blocking solution at 37°C for 5 min or 18°C–25°C for 15–30 min.

***Note:*** We have not found significant reduction in background staining with longer incubation times, but according to the manufacturer (Vector Laboratories), other users may have seen benefit from longer incubation times.

62. Wash 3 × 2 min each in PBST.
63. Seal plate around the edges with parafilm.
64. Incubate in primary antibody solution in SuperblockT at the indicated concentrations, shaking at 4°C for 1–7 days or 18°C–25°C 1–3 days.

***Note:*** Optimal incubation times will depend on the antibodies. Example images for this protocol use the primary antibodies noted in the key resources table (1:2000 GFAP, 1:500 Sox10, 1:500 HuCD).

65. Wash 3× 3–5 min each in PBST.
66. Incubate in secondary antibody solution at 37°C for ∼30 min or at 18°C–25°C for 40–55 min.

***Note:*** Secondary antibody selection will depend on primary antibody hosts. Example images for this protocol used 1:500 dilutions of Alexa Fluor dyes (i.e., Invitrogen Donkey anti-Chicken 488, Donkey anti-Mouse 555, Donkey anti-Gt 647; key resources table).

67. Incubate in Hoechst (nuclear stain) at 10 μg/mL in PBST for 5–10 min at 21°C.
68. Wash 3× at least 10 min each in PBST.
69. Briefly rinse tissue 3× with PBS.
70. Mount tissue on glass bottom dishes under a dissection microscope.

***Note:*** It is best to mount the thinner muscle layer side down, such that the myenteric plexus is closest to the glass for imaging. This requires focusing the image well under a dissection microscope.

**CRITICAL:** Make sure all the tissue segments stay moistened with PBS while mounting other segments. They can dry out quickly under the light of a hot dissection lamp. If they dry out, they may detach from the plate and be lost. They can be kept moistened using PBS in a 10 μL pipette.

71. Remove excess PBS with a pipette.
72. Immediately place a drop of Prolong Gold mounting medium to the center of the well.
73. Gently lay down a circular glass coverslip on the tissue to flatten.

**CRITICAL:** Ensure the mounting medium spreads across the glass to surround all tissues.

***Note:*** If there are large pockets of air that do not resolve after a few minutes as the mountant diffuses under the coverslip, additional mountant can be added with a 10 μL pipette to the edge of the coverslip.

**Pause point:** Allow the mounting medium to cure at 18°C–25°C shielded from light for 18–24 h before imaging.

74. Use a confocal microscope to take images of the myenteric plexus or other tissue area of interest.

***Note:*** Selection of objective size should be based on the specific needs of your study (i.e., to provide adequate tissue coverage and resolution within an image field.) A good starting point may be a 20× oil immersion objective.

***Note:*** If analyzing ganglia, aim to capture most of the area of an individual ganglion in the image. Keep in mind that ganglion sizes often differ between intestinal regions and species.

***Note:*** The choice of where in the tissue to image should be based on the analysis to be performed, e.g., to maximize the neuronal cell bodies within a single field.

***Note:*** Any confocal microscope with the capacity to image the wavelengths of your secondary antibodies should be adequate here.

***Note:*** Institution-specific training may be required before using microscopes in core facilities.

***Note:*** We recommend taking at least 3 non-overlapping images per stained tissue section.

**Pause point:** Label the files with sufficient detail for later reference (e.g., animal ID, antibodies, date).

***Note:*** When processing images later for analysis, it is recommended have someone help you blind the file names or to use a self-blinding script (e.g.,^10^).

***Note:*** Multi-channel images may be loaded into Fiji for further processing.

75. Load images as multichannel images in Fiji.
76. Pre-process images using Gaussian blur (with sigma = 0.7) and Background Subtract (rolling ball radius = 50 px) (Figure 8) (Optional).
77. Using the freehand selection tool, outline a ganglion (based on GFAP staining to delineate the ganglion border).

**CRITICAL:** Choose ganglia that contain at least 15 neuron soma.

78. Add the ganglion selection to ROI Manager.
79. Save the ROI.
80. Using the Cell Counter tool, count the number of clearly visible round nuclei within the ganglion outline.
81. Count the HuCD positive cells.
82. Count cells positive for a marker of interest – e.g., Sox10.
83. Save the markers in the Cell Counter tool.

***Note:*** We have found that counting cells by hand yields more accurate results than automated scripts. Whole-mount tissue irregularities make automated analysis scripts prone to errors.

***Note:*** GFAP staining can help to define the borders of the ganglion. We have defined ganglia as a cluster of HuCD positive cell bodies separated by no more than 1 cell body diameter distance from each other. To identify the delineating border of the ganglia, we found that GFAP staining seemed to encircle the neuronal soma closely while also including glial cells that formed part of the ganglion but that would have otherwise been cut out by defining the ganglion just by the neuronal soma.

**Figure 7:**
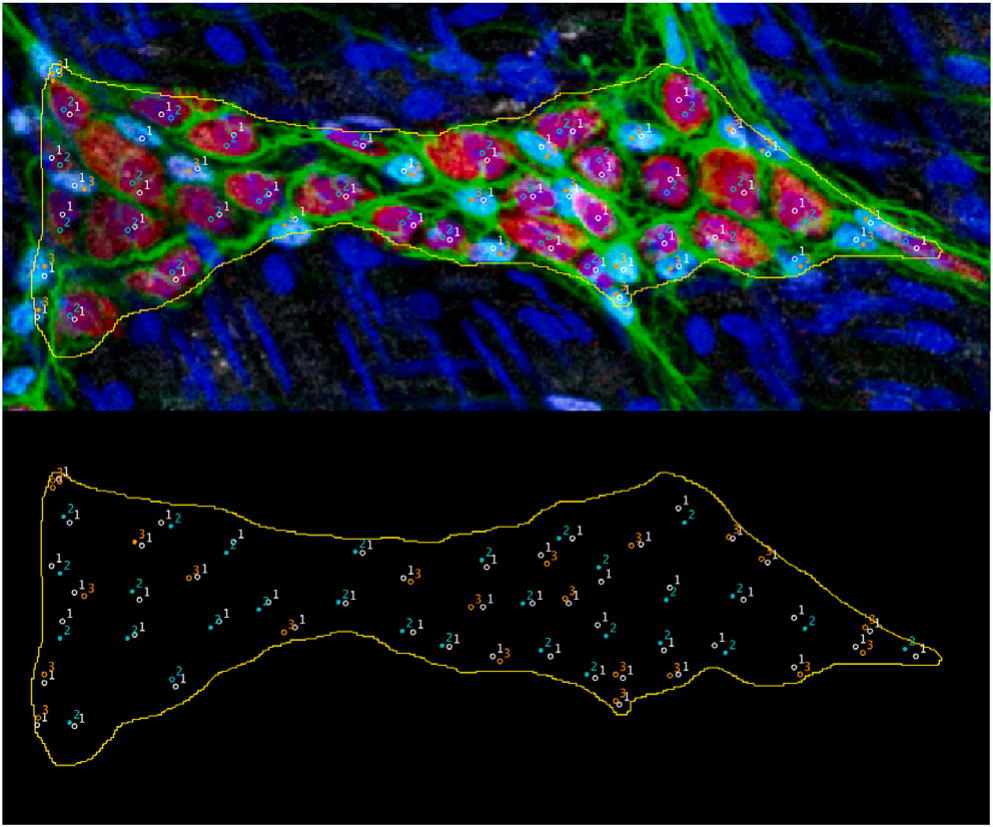
Example of the ganglion ROI and the counts for nuclei, neuronal soma, and Sox10+ glial cells Marking the nuclei first and then ensuring that all marked neurons and glia correspond to a nucleus can serve as an internal check to avoid overlooking cells within the ganglion. Top panel: Blue: Hoechst (nuclei). Red: HuCD/Elavl3,4. White: SOX10. Green: GFAP. Bottom panel: “1” marks nuclei; “2” marks neuronal soma; “3” marks SOX10+ glia. Yellow outline uses GFAP staining to assist with marking ganglion border.

***Note:*** Counting total nuclei within a ganglion can serve as an experimental check to ensure accuracy of counts and reduce errors: Hoechst staining allows comparison between nuclei counts and the sum of the cells labeled with cell markers of interest. If there are some nuclei that are negative for all markers of interest, helpful still to mark these as such. Figure 7 shows an example of the ganglion ROI and the counts for nuclei, neuronal soma, and Sox10+ glial cells.

## Expected outcomes

Transit times are expected to vary within animals of the same group and even within the same individual. In our experience, transit times have ranged from <3 h to >9 h, with the average time between 3-5 h. Taking an average of repeat measurements facilitates a more accurate representation of each animal’s motility. Additionally, wide differences in transit time are expected between germ-free (GF) and/or antibiotic treated mice compared to mice with a full set of microbes. Differences in transit times between male and female mice are also expected, as are their responses to many experimental manipulations.

**Figure 8:**
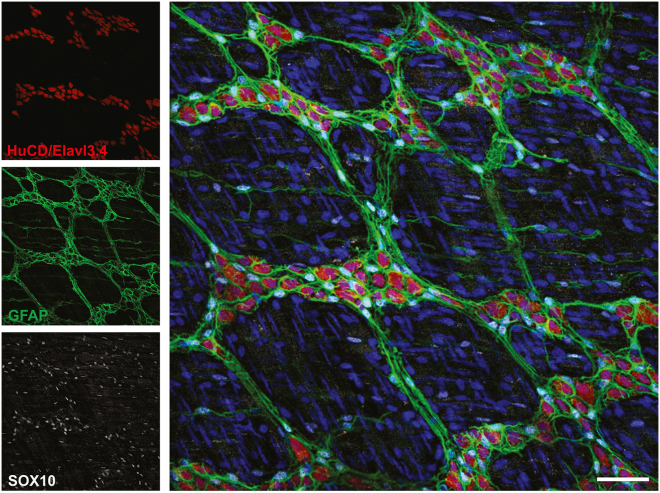
Whole-mount immunostaining of distal colon myenteric plexus Blue: Hoechst (nuclei). Red: HuCD/Elavl3,4. White: SOX10. Green: GFAP. Image processed in Fiji with Gaussian blur (sigma = 0.7) and Background Subtract (rolling ball radius = 50 px). Scale bar 50 um.

For image analysis, the readout in this case is proportions of cells within a ganglion that are positive for a particular marker. An example image is shown in Figure 8.

## Quantification and statistical analysis

### Record keeping

Transit testing data may be recorded in a lab notebook and transferred later to an excel sheet. The date, start and stop times should be indicated for each animal and the duration of transit time can be calculated. Record the number of pellets excreted during that period for each mouse. During image analysis, counts from Fiji should be recorded separately (e.g., a digital spreadsheet), as they may not reliably save in the program file.

### Transit testing averaging

Based on your longitudinal time points (i.e., based on age of the mouse, or time since manipulation, before/after, etc.), animals can be binned into the appropriate groups – for example, repeat trials from one mouse at age 11 weeks, 12 weeks, and 13 weeks might be averaged to yield one transit time value for that mouse at approximately 12w of age. The average transit time value for each animal at each time point can then be used to represent that animal’s motility at that time point.

### Transit testing study design

For longitudinal measurements, it may be wise to include an analysis that shows the results from animals when tested at the same age as well as another analysis that shows results from animals when tested at the same amount of time since the experimental manipulation. (For example, comparing ∼16-week-old mice given FMT at different times during development in one analysis and comparing ∼8-week-old and ∼16-week-old mice at the “4 weeks post FMT” time point.)

### Statistical analysis

Multi-group analyses of this type are often best done as a 1- or 2-way ANOVA with appropriate correction for multiple comparisons. Rather than comparing every group pairwise when all comparisons are not informative, a priori selection of the groups to be compared may provide more statistical power and greater sensitivity for meaningful differences between groups.

### Exclusion criteria

Criteria for excluding a mouse or trial from an analysis might include the case in which an animal’s first stool pellet during transit testing contained residual dye from a previous trial. (Waiting several days between trials reduces the likelihood of this happening.) Similarly, if a mouse is found to develop a significant health condition unrelated to the study protocol (e.g., malocclusion causing decreased food intake), that mouse can reasonably be excluded and not tested further. For image analysis, the decision to exclude images from the analysis set (e.g., due to faint or poor quality staining of one or more markers) should be made while blinded to group membership.

## Limitations

### Limitations of transit testing

• Several possible threats to validity
  ○ It is generally most practical for the experimenter to conduct transit testing during typical human waking hours – i.e., daytime – but the physiologic active times for a mouse are in darkness.^11^ Beginning transit testing around the same time for each experiment can mitigate against potentially inconsistent results based on time of day.
  ○ Transit time of an animal that is anxious may be decreased relative to its baseline – for example if there are unintended loud noises or other disruptions during testing. Testing the same mouse multiple times helps to decrease the impact of occasional minor disruptions to testing but if disruptions are frequent and present across repeated trials, the transit testing results may not represent the transit time of that mouse at its baseline.
  ○ Because mice are coprophagic, a mouse may eat the initial red pellet before the experimenter sees it, for example if the pellet sticks on the wire rack rather than falling to the bottom of the cage out of reach; if this occurs, the transit result for that animal may be artificially longer than it truly was. Keeping close watch on the animals – and testing a manageable number of mice at a time – reduces the likelihood of the experimenter missing the appearance of the first red pellet.
• Time and labor intensive: This protocol is time intensive, requiring frequent monitoring of animals; however, the results of this metric are one of the most relevant measurable physiologic indicators of motility in mice, making the experiments worth the time. Ideally, the same experimenter conducts transit testing across all groups, since subtle differences in handling of mice could potentially affect outcomes.
• Potential subjectivity: The subjective determination of what is “red” may differ slightly between experimenters, though we recommend establishing very clear criteria for what constitutes the first red pellet, as noted above.
• Logistical challenge with germ-free mice: Transit testing, particularly repeated transit testing, can be difficult to do with germ-free mice, although it is possible. It requires assistance coordinating the study with gnotobiotic facility animal caretakers.
• Not universally usable: This protocol may not be appropriate for neonatal or pregnant mice, for whom compression during scruffing the animal for gavage could be detrimental to the animal’s health.

### Limitations of whole-mount staining and image analysis

• Tissue stretch affects counts. If stretch is inconsistent, this could skew results of area-based calculations. We have avoided area-based calculations in this protocol, using relative nuclei counts to anchor other cellular counts.
• Subtle differences between groups may be difficult to detect with immunofluorescence analysis if image output is limited. Slight differences in intestinal regions between samples could affect cell proportions.
• Manual image analysis is time and labor intensive.

## Troubleshooting

### Problem 1

Difficulty identifying which pellets are “red” (longitudinal assessment of gastrointestinal motility).

### Potential solution

Collect pellets with forceps and smear onto paper towel. If color blindness is an issue, consider trying a different dye color such as Evans blue or charcoal.

### Problem 2

Transit testing results are highly variable, even within the same experimental group (longitudinal assessment of gastrointestinal motility).

### Potential solution

Variability in transit times is expected, both within the same experimental group and on repeat testing in individuals. As described in the protocol, repeat testing in the same individual after a few days is highly recommended to allow an average value to be computed for the individual.

### Problem 3

Tissue staining is absent (Whole-mount staining, imaging, and analysis of myenteric neurons and glia).

### Potential solution

Assuming that both the primary and secondary antibodies were added appropriately, this may be an issue of over-fixation or inadequate epitope retrieval. For tissues that remained in fixative for more than 1–2 days, it may be necessary to extend the epitope retrieval incubation time. Ensure that the tissue remains submerged in buffer during epitope retrieval and does not dry.

### Problem 4

Cells appear swollen on imaging (Whole-mount staining, imaging, and analysis of myenteric neurons and glia).

### Potential solution

The tissue may have been placed in a hypotonic solution, such as water, instead of PBS. Ensure that buffered solutions are used for all wash and incubation steps during staining.

### Problem 5

Tissue is curled and doesn’t lay flat for imaging (Whole-mount staining, imaging, and analysis of myenteric neurons and glia).

### Potential solution

Pin the tissue flat prior to fixation during tissue harvest. If the tissue was pinned flat but still has curled areas, trim off the curled areas or and/or cut small (few millimeters) sections that can be flattened by the glass coverslip.

### Problem 6

Tissue staining is faint (Whole-mount staining, imaging, and analysis of myenteric neurons and glia).

### Potential solution

This is often either an issue of epitope retrieval or inability of the antibody to bind with intestinal antigens. (The latter is less of a concern with the antibodies used here, but many other neuron-related antibodies have been validated in brain and not intestine.) Consider whether the staining times and antibody concentrations have been appropriate. Ensure the tissue is flat against the cover glass; this can be assessed by checking the Hoechst staining; if nuclei come into focus but not other markers, it is more likely an issue of staining, whereas if the nuclei do not come into focus, this could be an issue of the regions of interest being far from the cover glass (e.g., due to interfering tissue or due to not laying flat on the glass) or could be a tissue quality issue.

### Problem 7

High background staining and/or images are blurry (Whole-mount staining, imaging, and analysis of myenteric neurons and glia).

### Potential solution

Make sure the epithelial layer has been peeled off, as we have found that epithelial antigens can sometimes strongly cross react with certain secondary antibodies – make sure to include secondary antibody only controls and compare “signal” to any staining patterns seen in the negative controls. You may also consider extending the time for mouse-on-mouse blocking or rinsing secondary antibody longer. Additionally, consider trying citrate buffer rather than Tris-EDTA buffer given reports of less tissue damage with citrate buffer^12^; however, we have not observed significant differences in background staining between the two buffers.

## Resource availability

### Lead contact

Further information and requests for resources and reagents should be directed to and will be fulfilled by the lead contact, Eugene B. Chang (echang@bsd.uchicago.edu).

### Technical contact

Technical questions on executing this protocol should be directed to and will be answered by the technical contact, Mary Frith (maryefrith@uchicago.edu).

### Materials availability

No new materials were generated by this protocol.

### Data and code availability

No new code was generated by this protocol. Please see the associated *iScience* manuscript for access instructions for datasets generated during this study.

## Acknowledgments

Research reported in this publication was supported by the UChicago 10.13039/100012848DDRCC, Center for Interdisciplinary Study of Inflammatory Intestinal Disorders (10.13039/100000062NIDDK P30 DK042086). M.E.F. is supported by 10.13039/100000002NIH T32 GM007281 and 10.13039/100000002NIH F30DK126309. D.R.L. is supported by 10.13039/100000002NIH R01DK129315. P.C.K. is supported by 10.13039/100000002NIH R01DK114007. The content is solely the responsibility of the authors and does not necessarily represent the official views of the NIH.

The authors thank the UChicago Functional Genomics and Integrated Light Microscopy Core Facilities, Gnotobiotic Research Animal Facility and Animal Resources Center, and Argonne National Laboratory Environmental Sequencing Facility. Biorender.com was used to create Figures 1, 2, 3, 4, 5, and 6 and portions of the graphical abstract.

## Author contributions

M.E.F. wrote the manuscript and carried out the protocol described herein. E.B.C. funded the work associated with this manuscript and provided expert advice. P.C.K. and D.R.L. contributed methodological expertise and advised on the protocols described by this manuscript.

## Declaration of interests

The authors declare no competing interests.
